# Angiopoietins lack of prognostic significance in ductal mammary carcinoma

**DOI:** 10.1186/1477-7800-4-6

**Published:** 2007-03-23

**Authors:** Khaled A Rmali, Gareth Watkins, Antonio Douglas-Jones, Robert E Mansel, Wen G Jiang

**Affiliations:** 1 Department of Surgery, Cardiff University School of Medicine, Cardiff, UK; 2 Department of Pathology, Cardiff University School of Medicine, Cardiff, UK

## Abstract

Angiopoietins (Ang) have been shown to regulate the process of vasculature and angiogenesis in tumour. Different angiopoietins have different roles during the angiogenic process. The current study sought to examine the levels of the expression of Ang-1, Ang-2, Ang-3 and their receptor Tie-2 in mammary ductal carcinoma and to assess their relevance to prognosis. Fresh frozen ductal carcinoma tissues (n = 90) and adjacent non-cancerous breast tissues (n = 32) were used. The expression of Ang-1, Ang-2 and Ang-3 transcripts in cancer and normal breast tissues were examined quantitatively using quantitative RT-PCR. The protein expression of Ang-1, Ang-2 and Tie-2 was assessed by immunohistochemistry on frozen sectioned tissues.

Ang-1, Ang-2 and Ang-3 were detected in mammary tissues. Ang-1 was seen in both normal epithelial cells, breast cancer cells as well as in endothelial cells. Ang-2 was seen at a higher level than Ang-1 and it is expressed in epithelial, endothelial as well as stromal cells to certain degree. Ang-1 and Ang-2 transcripts were detected almost equally in cancer and normal breast tissue, and Ang-3 was high in cancer tissue compared to normal breast but not significant (155 ± 123 & 24.1 ± 22.6, P > 0.05). No significant differences were seen between patients with different predicted prognosis (using the Nottingham Prognostic Index as a guide) (Ang-1 p = 0.34, Ang-2 p = 0.26 and Ang-3 p = 0.32, respectively). No significant correlation was seen between Ang-1, Ang-2 and Ang-3 with tumour grade. When the levels of the transcripts were compared against clinical outcome (disease free, developed recurrence and patients who died of breast cancer), levels of Ang-3 transcript was found to be high in breast cancer patient who had bone metastasis 33.8 ± 28.3, although the difference was not significant (p = 0.08). No significant difference was seen with levels of Ang-1 and Ang-2 transcripts and clinical outcomes. Furthermore, no significant trend was observed between Tie-2 receptor and clinical/pathological parameters in the cohort.

These data suggest that angiopoietins (Ang-1, Ang-2 and Ang-3) are expressed in mammary tissues, both in normal and tumour. These molecules have limited value in predicting the prognosis and clinical outcome in patients with mammary ductal carcinoma.

## Background

Angiogenesis, generation of new microvessels from pre-existing blood vessels, is essential for tumour growth and invasion [1]. Cancer cells stimulate angiogenesis by secreting angiogenic factors, such as vascular endothelial growth factor (VEGF), platelet derived growth factor (PDGF), and fibroblast growth factor (FGF) [2]. Angiopoietins, a new family of angiogenic growth factors that are mostly specific for the vascular endothelium, have been identified in recent years [3,4]. Ang-1 plays a role in maintaining and stabilizing mature vessels by promoting the interaction between endothelial cells and surrounding support cells, whereas Ang-2 is expressed at sites of vascular remodelling and is thought to antagonise the stabilising action of Ang-1 [5,6]. Ang-1 and Ang-2 share about 60% amino acid identity and bind with similar affinity to the endothelial cell tyrosine kinase receptor Tie2 [4,7]. Angiopoietins and Tie-2 are related to vascular remodelling and sprouting, which occur in a complementary and coordinated fashion during vascular development, along with vascular endothelial growth factor (VEGF) and its tyrosine kinase receptors (Flk-1 and Flt-1) [8].

Breast cancer is the most common form of malignancies in females in the U.K, and metastasis of breast cancer is common. About 7% of patients with breast cancer present with widespread metastases at the initial presentation [9]. The most common sites of metastasis are bone, lungs, liver, chest wall and central nervous system. Less common sites are the adrenals, ovaries, pericardium, thyroid and bone marrow [10]. Tumour cell dissemination is mediated via a number of mechanisms, including local tissue invasion, haematogenous and/or lymphatic spread as well as direct seeding of surfaces or body cavities. Angiogenesis plays a pivotal role in the vascular spread of breast cancer as well as key to the growth of breast tumours.

Up-regulation of angiopoietins expression has been noted in many malignant cancers such as gastric carcinoma [11,12], colorectal cancer [13], hepatocellular carcinoma [14], renal cell carcinoma [15], ovarian cancer [16] and non-small lung cancer [17]. Although a few reports on the expression of angiopoietins in breast cancer have become available in recent years [18,19], these studies provide little conclusive evidence of the correlation between these molecules and breast cancer progression.

In the present study we examined the expression Ang-1, Ang-2 and for the first time Ang-3 and their receptor Tie-2, at mRNA and protein levels in human ductal mammary carcinomas and investigated the correlation between the level of the expression of these molecules and clinical/pathological parameters of breast cancer.

## Materials and methods

### Tissue collection and preparation

Ductal cancer tissues of the breast (*n *= 90) were collected immediately after surgery and stored in a deep freezer until use. 'Normal' (adjacent non-cancerous) (*n *= 32), which were from the same patients with breast cancer and away from tumour margins were also collected. Patients were routinely followed clinically after surgery as previously described [20]. The median followup of the cohort was 120 months.

### Tissue processing, RNA extraction and cDNA synthesis

Total RNA was isolated from frozen-sectioned breast tissues ('normal' (adjacent non-cancerous) and cancer). Multiple sections were homogenised to extract RNA, using RNA-Zol reagent (ABgene, Epsom, Surrey, UK), according to manufacturer's instructions, as we previously reported [21]. cDNA was generated from 1 μg RNA using an AMV-reverse transcription kit (ABgene, Epsom, Surrey, UK), and the standard gunanidine isothiocyanate method as described in the manufacturer's protocol. The purity and concentration of RNA was determined by spectrophotometry at 260 nm and 280 nm.

Conventional PCR primers were designed using Beacon Designer software (Palo Alto, California, USA), to allow amplification of regions that have no overlap with known genes and span at least one intron. Primers were synthesized by Life Technologies (Paisley, Scotland, UK) (Table 1).

**Table 1:** Primer sequence.

	**Sense primer F1(5'-3')**	**Antisenes primer ZR(5'-3')**
**Ang-1**	ttctcttcccagaaacttca	actgaacctgaccgtacacatctccgactt
**Ang-2**	tcatggaaaacaacactcag	Actgaacctgaccgtacattctgtactgcattctgctg
**Ang-3**	Gtggctgaagaagctagaga	Actgaacctgaccgtacagtctgattctgggccatt
**Tie-2**	Acaacatagggtcaagcaac	actgaacctgaccgtacagatggctataa

### Quantitative analysis of the Ang-1, Ang-2, Ang-3 and Tie-2 transcripts in tumour and normal breast Tissues

We employed the iCycler IQ system (BioRad, Camberley, UK), to quantify the transcript level (shows as copies/μl calculated from internal standard) of the Ang-1, Ang-2, Ang-3 and Tie-2, as we previously reported [20]. The system used a universal probe (UniPrimer™), which recognised a specific sequence (z sequence), which had been incorporated into the primers. The reaction was carried out using IcyclerIQ™ (Bio-Rad) which is equipped with an optic unit that allows real time detection of 96 reactions, using the following condition: 94°C for 12 min, 50 cycles of 94°C for 15 s, 55°C for 40 s and 72°C for 20 s. All samples were simultaneously examined for Ang-1, Ang-2, Ang-3 and Tie-2 along with appropriate set of plasmid standards and negative controls. Primers sequences used for Ang-1, Ang-2, Ang-3 and Tie-2 were shown in Table 1.

### Immunohistochemistry

This was as we recently reported [22]. Briefly, immunohistochemical staining was performed on paired frozen-sectioned tissues (cancer tissue paired with normal background tissue from the same patient). Frozen sections were cut, air dried and fixed in 50% methanol and 50% acetone for 15 minutes. The sections were then air dried once more for 10 minutes and stored at -20°C in foil-wrapped slide trays. Immediately before staining specimens were then placed in PBS (Optimax wash buffer) for 5 minutes. The slides were incubated with primary rabbit polyclonal antibodies against Ang-1, Ang-2 and Tie-2 (Santa Cruz Biotechnologies Inc., Santa Cruz, CA, USA) or positive control at 1:50 dilution for 1 hour. After 4 washes with PBS, the slides were placed in universal multi-link biotinylated (Dako). Secondary antibody at (1:100) dilution and incubated for 30 minutes. This was followed by 4 washes with PBS. Slides were then placed in avidin biotin complex (ABC – Vector Labs) for 30 minutes. The bound antibody complex was detected using diaminobenzidine tetrahydrochloride (3,3'-diaminobenzidine)-DAB (Sigma), chromogen for 5 minutes. The slides were washed with H_2_O for 5 minutes and placed in Mayer's haematoxylin for 1 minute, followed by differentiation in H_2_O for 10 minutes. This was followed by dehydration in methanol (3 times) and clearing in 2 changes of xylene before mounting under cover slip and examined on the microscope negative controls (using PBS buffer instead of the primary antibody) and positive controls were used in this study. The complete procedure was carried out at room temperature.

### Statistical analysis

Statistical analysis was carried out using Minitab version using a Macro written for the study. Statistical analysis was performed using a Student's t-test.

## Results

### The level of the expression Ang-1, Ang-2, Ang-3 and Tie-2 in tumour and normal background breast tissues and in relation to node status

**Figure 1:**
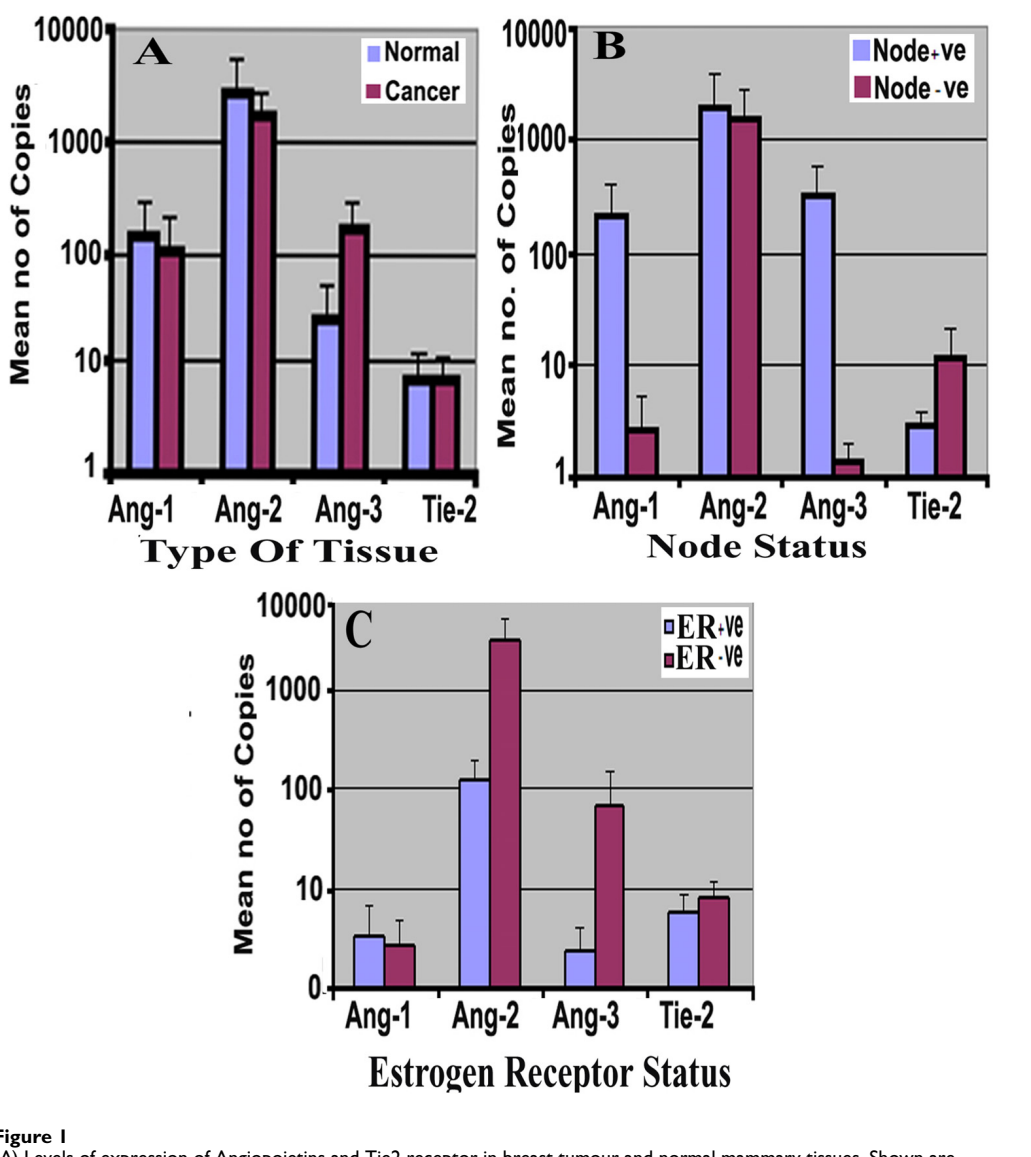
(A) Levels of expression of Angiopoietins and Tie2 receptor in breast tumour and normal mammary tissues. Shown are number (mean ± SD) of copies of respective transcripts from 50 ng total RNA. (B) Node negative tumours and node positive tumours had different levels of the Angiopoietins and Tie2 receptor transcript. Ang-1 and Ang-3 shown higher levels of copies in +ve node in contrast to Tie-2 receptor showed higher transcripts level in -ve node tumour. Both statistically not reached significant P > 0.05. (C) Angs/Tie2 and ER status. There was no significant difference in level of expression of all Angs and Tie-2 breast cancer tissue with/without Estrogens receptor P.0.05.

Ang-1, Ang-2, Ang-3 and Tie-2 transcripts were evaluated using quantitative PCR. The Ang-1 transcript level in background tissues was marginally higher than in tumour tissues (mean ± SD 141 ± 135 vs 101 ± 100, for background and tumour respectively, p > 0.05) and Ang-2 (2777 ± 2691 vs 1686 ± 1049 p > 0.05). In contrast, Ang-3 showed higher level in tumour compared to background tissue (tumour 155 ± 123 vs background: 24.1 ± 22.6, P > 0.05) (Figure 1A). Similarly, no significant difference was seen between node negative and node positive tumours for Ang-1, Ang-2 and Ang-3 transcript (p > 0.33, p > 0.83 and p > 0.21 respectively) (Figure 1B).

The level of the Tie-2 receptor showed no significant difference between tumour and background (tumour: 7.1 ± 4.7 vs background: 7.1 ± 7.4, P = 0.87). Although there were marginally higher levels of the Tie-2 receptor in node positive tumours, this did not reach significance (median value node positive: 11.5 *vs *median value node negative: 2.9 P = 0.37).

Oestrogen receptor positive breast tissues (ER ve+) had low level of Ang-2 and Ang-3 compared to ER negative tumours (ER ve-) (ERve+ for Ang-2: 120 ± 75, Ang-3: 2.4 ± 1.6 *vs *ERve- for Ang-2: 2970 ± 1973 & for Ang-3: 65 ± 85). Ang-1 showed higher levels of transcripts in ERve+ tumours (3.4 ± 3.2), compared with ERve- (2.6 ± 2.3). Again, the differences were not significant p > 0.05 (Figure 1C).

### Correlation of Ang-1, Ang-2, Ang-3 and their receptor Tie-2 with prognosis and staging

The Nottingham Prognostic Index (NPI) was used as one parameter to assess the prognosis of the patients, in that patients with NPI value less than 3.4 (NPI-1) were regarded as with good prognosis, 3.4–5.4 (NPI-2) moderate prognosis, and NPI>5.4 (NPI-3) with poor prognosis. The formula for NPI being: NPI = [0.2_size cm] + grade (1 - 3) + nodal status (1 - 3).

**Figure 2:**
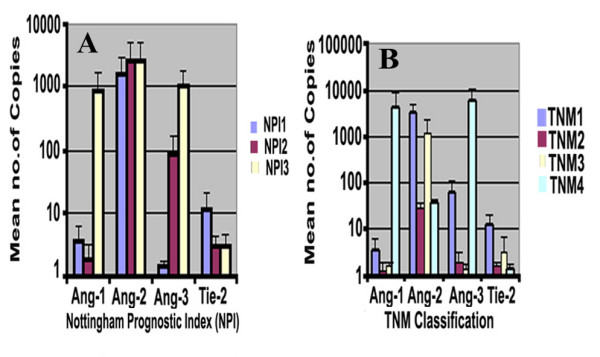
(A) Quantitative polymerase chain reaction (Q-PCR) analysis of breast cancer tissue samples showing higher transcript levels of Ang-1 and Ang-3 in poor prognostic index NPI-3 (p > 0.05), and almost similar levels of Ang-1 and Tie2 transcripts in all three groups. (B) Advanced stage of breast cancer TNM4 showed higher, but insignificant, levels of Ang-1 and Ang-3.

There were higher levels of Ang-1, Ang-2 and Ang-3 expression seen in patients with poor prognosis, i.e., NPI-3 tumours (NPI >5.4), although this did not reach significance (for Ang-1 NPI-1: 3.69 ± 2.5; NPI-2: 1.8 ± 1.4; NPI-3: 806 ± 805; P = 0.34) (for Ang-3: NPI-1 0.38 ± 0.28; NPI-2: 85.7 ± 84.3; NPI-3: 1000 ± 961; P = 0.32). Higher levels of Ang-2 were observed in moderate prognosis, i.e. NPI value at 3.4–5.4 (NPI-2) (NPI-1: 1510 ± 1282; NPI-2: 2641 ± 2372; NPI-3: 50.6 ± 23; P = 0.32) (Figure 2A). The levels of the molecules were also analyzed against TNM status. No significant difference was seen between any groups for all three Angs (Figure 2B).

No trend was observable among levels of the angiopoietin receptor Tie-2 receptor expression and NPI (NPI-1: 11.5 ± 9.4; NPI-2: 2.9 ± 1.1 p > 0.36; NPI-3: 2.9 ± 1.5; P > 0.37) or with TMN status.

### Angiopoietin -1, -2, -3 and Tie-2 expressions in different tumour grade

**Figure 3:**
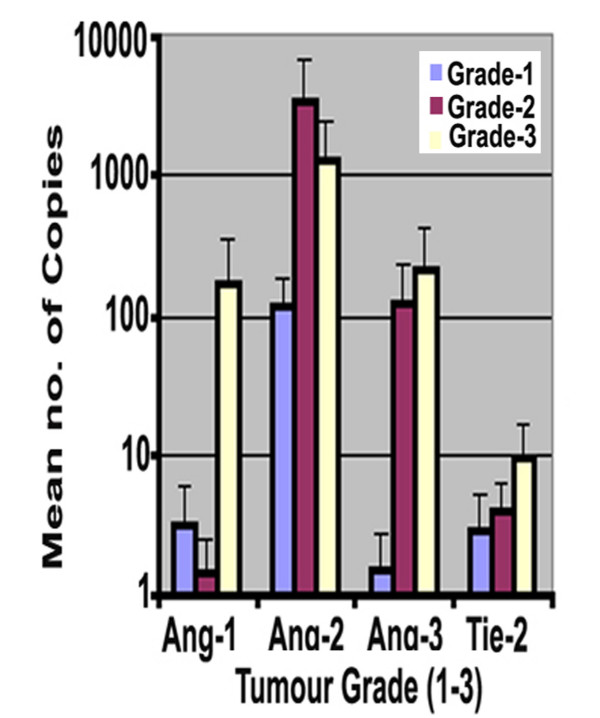
Angs and Tie2 transcript and tumour grade. Grades 3 breast tumours demonstrate higher levels of Ang-1, Ang-3 and Tie-2 compared to Grade 1. Ang-2 transcripts level raised in grade 2 breast tumours.

The results showed that Ang-1 and Ang-3 expression were increased with higher grade of tumour, i.e. Grade-3 (173 ± 170 & 212 ± 108, respectively) compared with Grade-1 (3.2 ± 2.9 & 1.5 ± 1.3, for Ang-1 and Ang-3 respectively) (Figure 3). The differences however were not statistically significant (p > 0.05). The level of Ang-2 transcripts was higher in Grade-2 tumours compared to Grade -1 (Grade2: 3520 ± 3301 vs Grade 1: 114 ± 69.6). There was little difference in the level of Tie-2 receptor expression between the grades of the breast cancers (Grade-1: 2.9 ± 2.1; Grade-2: 4.1 ± 2.3; Grade-3: 9.3 ± 7.5).

### Expression of Ang-1, Ang2, Ang-3, Tie-2 and clinical outcome following 10-year follow-up

**Figure 4:**
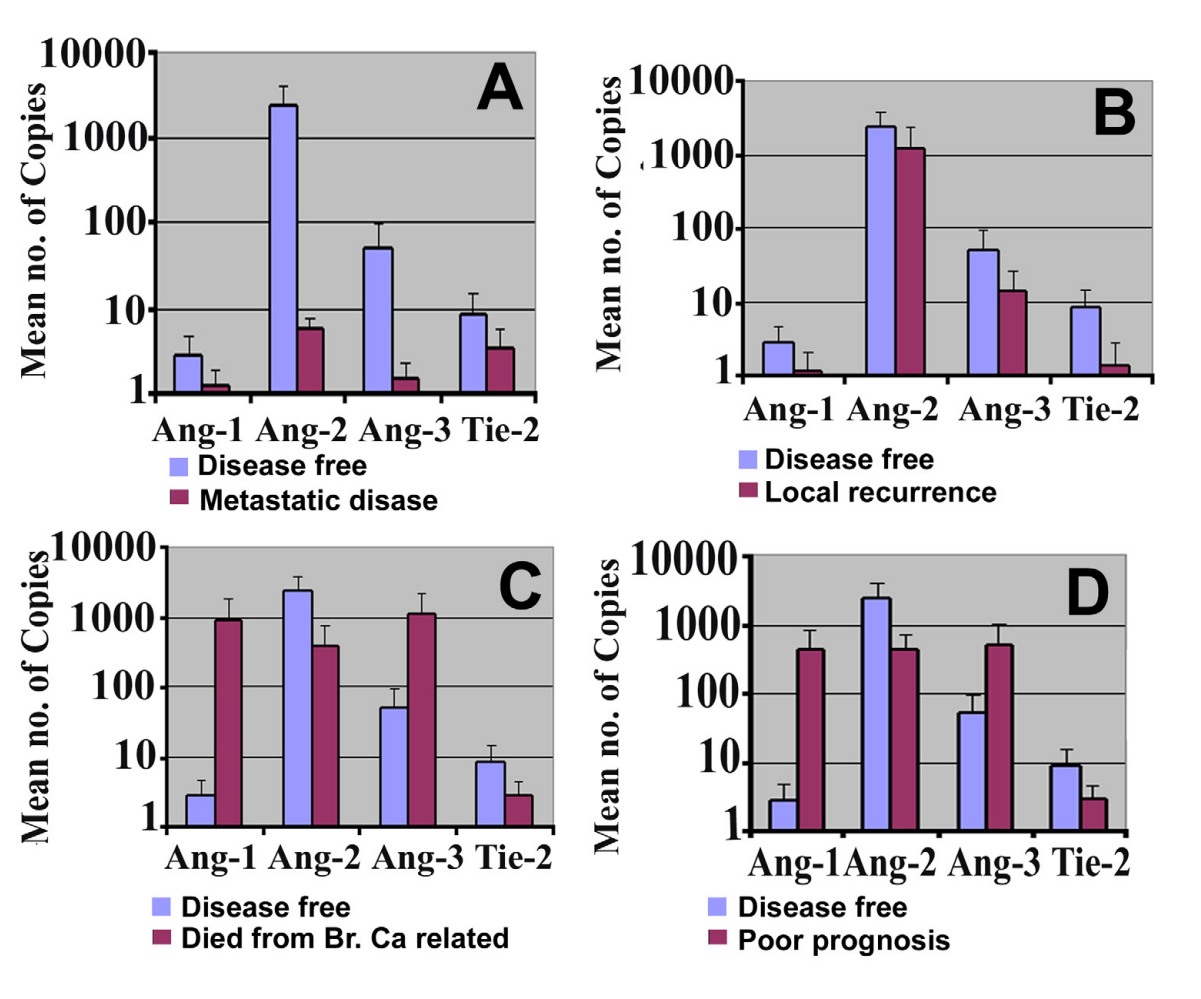
Q PCR analysis shows association between Angiopoietins and clinical outcome in breast cancer. (A&B): Patients who developed metastatic disease and local recurrence have low levels of Angs and Tie-2 transcripts than who diseases free. (C): Patients who died from breast cancer showed higher levels of Ang-1and Ang-3 copies than who diseases free (p > 0.05), whereas Ang-2 and Tie-2 had higher levels of expression in diseases free compared who died from breast cancer (p > 0.05). (D): Comparison between patients who remained disease free and those who developed breast cancer related progression (recurrence, metastasis and mortality). Ang-1 and Ang-3 transcripts had higher levels in poor prognosis patients in relation to diseases free patients, and again Ang-2 and Tie-2 transcripts had higher levels of expression in diseases free compared to poor prognosis. Neither reached a statistical significance.

Figure 4 shows data comparing patients with different clinical outcomes after a median follow-up of 120 months. Patients were divided into those who remained disease free, those who developed metastasis or local recurrence, or those who died of breast cancer. Those patients with metastatic disease had low, but insignificant levels of Ang-1, Ang-2, Ang-3 and Tie-2 expression compared to patients who remained disease free (metastatic group: Ang-1: 0.22 ± 0.16, Ang-2: 0.73 ± 1.8, Ang-3: 0.44 ± 0.42, Tie-2: 3.3 ± 2.5 vs disease free: Ang-1: 2.75 ± 1.9, Ang-2: 2314 ± 1525, Ang-3: 50.7 ± 44.5, Tie-2: 8.5 ± 6.5 respectively) (Figure 4A). Moreover, patients with local recurrence show lower levels of Ang-1,-2,-3 and Tie-2 expression when compared with patients who remained disease free (Figure 4B). Although patients who died of breast cancer had higher levels of Ang-1 (886 ± 876) and Ang-3 (1060 ± 1055) compared to those patients who were disease free, this was not statistically significant (P = 0.34 & p = 0.37, respectively). In contrast, Ang-2 and Tie-2 had lower levels of transcripts in patients died from breast cancer (Figure 4C).

When we combined the three groups (with metastasis, recurrence, and mortality) to form a poor prognostic group (referred to as Poor Prog. in Figure 4D) and compared this group with those who remained diseasefree (Figure 4D), higher levels of all Angiopoietins molecules were found in the patients with poor prognosis (Ang-1 422 ± 421, Ang-2 416 ± 268, Ang-3 508 ± 502) although this did not reached a significance.

### Immunostaining of mammary epithelial cells and breast cancer cells for Ang-1, Ang-2 and their receptor Tie-2

**Figure 5:**
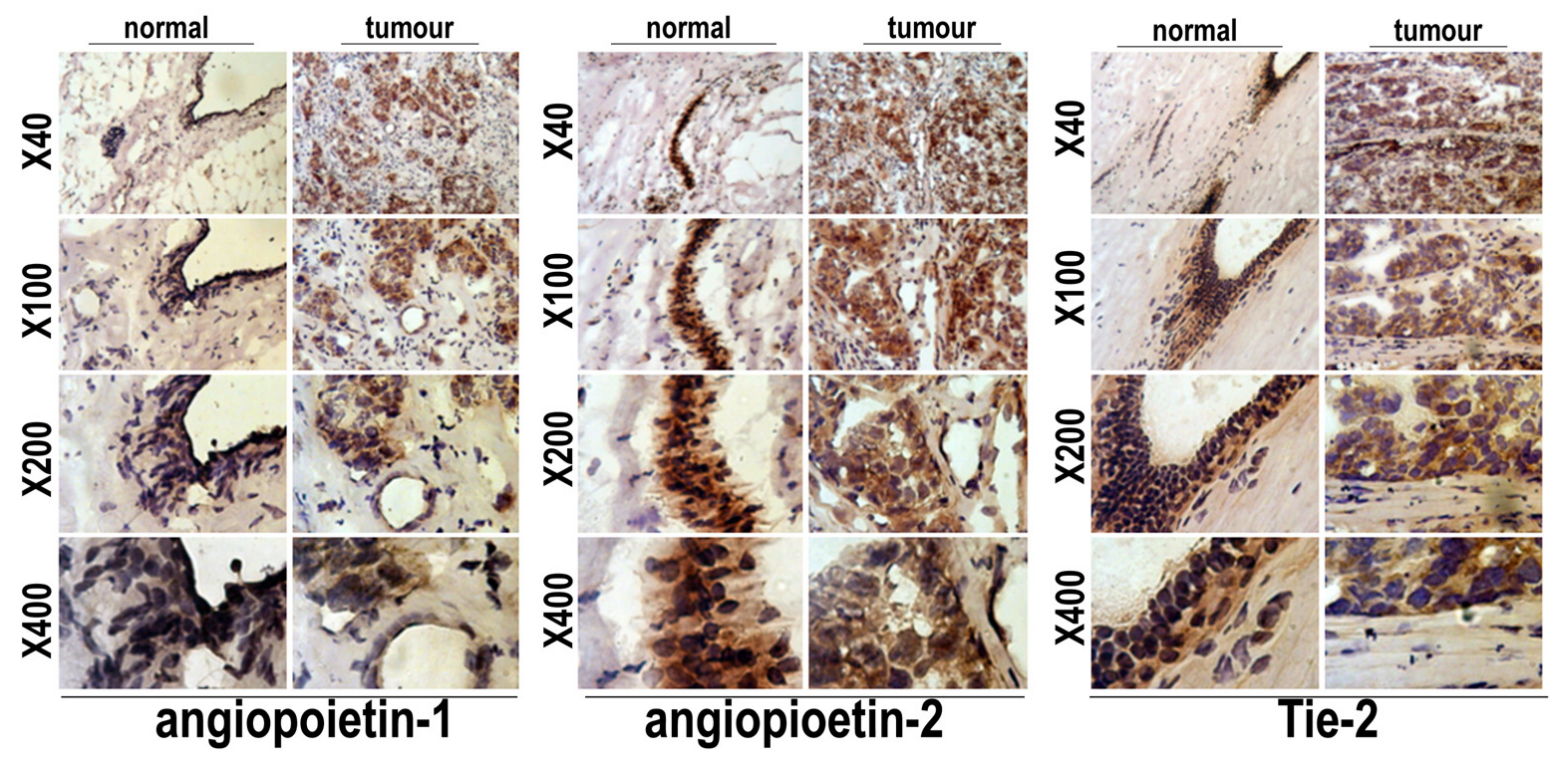
Immunostaining of normal background breast tissue (left Panel) and breast cancer tissue (Right Panel) for Ang-1, Ang-2 and Tie-2 (original magnification, 40×, 100×; 200× and 400×). A higher intensity of staining was noted in epithelial cells of breast cancer compared with non-cancerous mammary tissue. Strong staining of Ang-1, Ang-2 and Tie-2 is shown in normal breast duct compared to breast cancer duct.

Figure 5 shows the intensity of staining for Ang-1, Ang-2 and their receptor Tie-2 in tumour and background tissues. Stromal cells of normal mammary tissue showed weak staining of Ang-1, Ang-2 and Tie-2 compared with breast cancer. While the normal breast tissues duct showed strong staining of Ang-1, Ang-2 and Tie-2 compared with breast cancer duct epithelial cells.

## Discussion

Tumour metastasis and rapid growth is critically dependent on blood or lymphatic vessels routes. Prognosis of solid tumours are closely linked to angiogenic or lymphangiogenic factors. Despite numerous studies examining the angiopoietins in breast tumours, to our best knowledge the current study is the first study reporting the relationship of all three angiopoietins and Tie-2 with clinical/pathological of ductal carcinoma of the breast. The current study has reported that the expression of Angs and Tie-2 were almost similar in ductal carcinoma and normal background tissues, with the exception of Ang-3 which showed higher levels of expression in breast cancer compared with normal breast tissues.

In the current study, we failed to detect a significant difference when a number of clinical and pathological factors were considered. Furthermore, all three angiopoietins showed a marginal higher level of the respective transcripts in advanced breast cancer (NPI3 & Grade-3), although this did not reach statistical significance. Our findings are consistent with Currie *et al *[18] who showed no significant correlation between Tie2 (or the Angs) with most clinic/pathological indices in breast cancer. Although their study demonstrated a significant correlation between oestrogen receptor (ER) status and both Tie2 and Ang-4 expression, our results did not show this correlation which is in agreement with Tautsui et al [19].

It is noteworthy from the present study that low levels of Angs and their receptor Tie-2 were found in patients who had cancer metastasis and local recurrence compared to diseases free after 10 years fellow up. Patients who died from breast cancer had higher levels of only Ang-1 and Ang-3. These findings are in contrast with Sfiligoi et al. [23] who reported that Ang-2 associates with tumour aggressiveness, whereas Ang-1 does not. Moreover, previous studies have demonstrated that the expression of Angs has a strong prognostic significance in breast cancer and correlates with MVD and VEGF expression [19,23], while Currie et al. [18] found no significant relation between Ang-2 expression and MVD in breast cancer. One of the reasons for these discrepancies may be the difference in methodologies and the sample size used in the respective studies. The current study was limited by its sample number. A study with bigger cohort will certainly help to further clarify the observations.

Despite some studies show a significant correlation between Angs expression and clinic/pathological parameters in cancers other than breast cancer including gastric, liver and colorectal cancer [11,12,14]. It is clear from the present study and indeed other limited reports [18] that the case in ductal carcinoma of the breast is far from being clear. A number of possibilities exist. Firstly, different organs may have a difference vasculature network. In earlier studies examining the mechanism of human breast cancer angiogenesis, it has been demonstrated that endothelial cell proliferation is a relatively rare event in breast cancer [24, 25]. Secondly, breast cancer is a heterogeneous disease whose clinical outcome encompasses a wide spectrum of possibilities from definitive cure to early death. Axillary nodal status has been shown to be one of the most important prognostic factors in the patients, with up to 30% of node-negative patients eventually relapse.

Taken together, although angiopoietins have been shown to be expressed at higher levels in GI tumours, the same has not been seen in breast cancer as reported here. It is possible therefore that angiopoietins and Tie2 play a lesser role in the progression of breast, compared with other angiogenic factors and indeed lymphangiogenic factors as widely reported. In summary, although our study shows high levels of Angs mRNA in advanced breast tumour such as in Grade-3 and NPI3 compared with early stage of cancer (Grade-1&NPI1), no significant difference is obtained. We conclude therefore that these molecules may not be significant prognostic factors in ductal carcinoma of the breast. The observation warrant further studies in larger cohorts.
